# Nucleosome assembly protein 1 like 1 (NAP1L1) promotes cardiac fibrosis by inhibiting YAP1 ubiquitination and degradation

**DOI:** 10.1002/mco2.348

**Published:** 2023-08-15

**Authors:** Tianyu Li, Zhihui Niu, Tong Yu, Jinrui Li, Xin Lu, Mengqin Huang, Qianqian Wang, Xiaojiang Yu, Jiayue Feng, Bingqian Xu, Danyang Bing, Xuelian Li, Lifang Lu, Haihai Liang, Rui Yang, Bin Wang, Hongli Shan

**Affiliations:** ^1^ Department of Pharmacology (State‐Province Key Laboratories of Biomedicine‐Pharmaceutics of China, Key Laboratory of Cardiovascular Research, Ministry of Education), College of Pharmacy Harbin Medical University Harbin China; ^2^ Shanghai Frontiers Science Research Center for Druggability of Cardiovascular noncoding RNA, Institute for Frontier Medical Technology Shanghai University of Engineering Science Shanghai China; ^3^ Department of Basic Medicine, The Centre of Functional Experiment Teaching Harbin Medical University Harbin China; ^4^ Research Unit of Noninfectious Chronic Diseases in Frigid Zone (2019RU070) Chinese Academy of Medical Sciences Harbin China; ^5^ Department of Pharmacology, School of Basic Medicine Inner Mongolia Medical University Hohhot China; ^6^ Department of Cardiovascular Ultrasound, Zhongnan Hospital of Wuhan University Wuhan University Wuhan China

**Keywords:** cardiac fibrosis, myocardial infarction, NAP1L1, ubiquitination, YAP1

## Abstract

Myocardial fibrosis post myocardial infarction (MI) is characterized by abnormal extracellular matrix (ECM) deposition and cardiac dysfunction could finally develop into serious heart disease, like heart failure. Lots of regulating factors involved in this pathological process have been reported while the specific mediators and underlying mechanisms remain to need to be further investigated. As part of the NAP1 family, Nucleosome assembly protein 1 like 1 (NAP1L1) is expressed in a wide variety of tissues. Here, we report that NAP1L1 is a significant regulator of cardiac fibrosis and is upregulated in ischemic cardiomyopathy patient hearts. Enhanced expression of NAP1L1 can promote cardiac fibroblasts (CFs) proliferation, migration, and differentiation into myofibroblasts. In contrast, loss of NAP1L1 decreased fibrosis‐related mRNA and protein levels, inhibited the trans‐differentiation, and blunted migration and proliferation of CFs after Transforming Growth Factorβ1（TGF‐β1）stimulation. In vivo, NAP1L1 knockout mice enhanced cardiac function and reduced fibrosis area in response to MI stimuli. Mechanically, NAP1L1 binding to Yes‐associated protein 1 (YAP1) protein influences its stability, and silencing NAP1L1 can inhibit YAP1 expression by promoting its ubiquitination and degradation in CFs. Collectively, NAP1L1 could potentially be a new therapeutic target for various cardiac disorders, including myocardial fibrosis.

## Graphical abstract

Nucleosome assembly protein 1 like 1 (NAP1L1), which was the dysregulated hub protein in myocardial fibrosis post‐myocardial infarction. NAP1L1 binds to Yes‐associated protein 1 (YAP1) and influences its transcriptional activity. When knockdown of NAP1L1 can inhibit the protein expression level of YAP1 by promoting its ubiquitination and degradation in cardiac fibroblasts. Taken together, NAP1L1 is a master‐induced factor of fibroblast activation and myocardial fibrosis, which could be a potential hub for targeting the treatment of the disease.

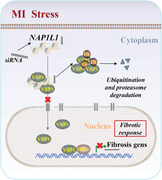

## 1 INTRODUCTION

Cardiac fibrosis is the most common feature involved in various kinds of heart diseases, and the primary cause of death across the world.^1^ The term always refers to an increased amount of extracellular matrix (ECM) in the tissues.^2^, ^3^ ECM is mainly produced by cardiac fibroblasts (CF),^4^ and activated CFs can transdifferentiate into myofibroblasts to secrete more collagen and other ECM‐related protein. Meanwhile, the molecular mechanism of cardiac fibrosis remains unknown and still needs further research.

Nucleosome assembly protein 1 like 1 (NAP1L1) is a member of the NAP1 family,^5^ which was identified in Xenopus laevis^6^ and is involved in many kinds of the biological process including nucleocytoplasmic shuttling, chromatin assembly, and remodeling, DNA replication and transcription.^7^ The human NAP1‐Like family consists of six subtypes (NAP1L1–6)^8^; among these, NAP1L1 and NAP1L4 are ubiquitously observed in many tissues, while others are expressed in brain tissues.^9^ As a histone chaperone, NAP1L1 can interact with the chromatin remodeler family CSB to enhance the CSB‐mediated chromatin remodeling process.^10^ It can also associate with H2A‐H2B dimers to regulate the transcription mechanism.^11^ As reported, NAP1L1 can modulate the acetylation of transcription factor p53 to regulate cell fate.^12^ Recently, a growing body of evidence has indicated that NAP1L1 is closely linked to cancer development.^13^ However, its role in cardiovascular diseases remains unknown.

Yes‐associated protein 1 (YAP1) is a transcriptional coactivator regulating cell proliferation, differentiation, apoptosis, and other physiological and pathological processes.^14^ YAP1 is also a vital effector of Hippo signaling which plays a crucial role in the response of fibroblast to Transforming Growth Factorβ1(TGF‐β1).^15^ Increasing evidence showed that activation of YAP1 exerts its function by interacting with DNA‐binding proteins, among which transcriptional coactivator with PDZ‐binding motif (TAZ) to promote the trans‐differentiation of CFs to myofibroblasts.^16^ Our findings showed that NAP1L1 can regulate cardiac fibrosis by modulating CF proliferation, migration, and trans‐differentiation by regulating YAP1. Posttranslational modifications like phosphorylation and ubiquitination are important in determining YAP1 activation or subcellular localization in various diseases.^17^ In this study, we found that NAP1L1 can promote YAP1 ubiquitination and degradation to regulate the cardiac fibrosis process.

NAP1L1 was identified for the first time in this study as a mediator of myocardial fibrosis. In ischemic cardiomyopathy (ICM) patients' hearts and myocardial fibrosis after MI, we detected higher expression of NAP1L1. More importantly, knockdown NAP1L1 restored cardiac function post‐myocardial infarction (MI) and inhibited excessive deposition of ECM, and blocked the fibroblast‐to‐myofibroblast trans‐differentiation process, indicating that it can be a potential treatment target for myocardial fibrosis. At the same time, we assumed that NAP1L1 had a significant role in regulating cardiac fibrosis by targeting the protein degradation process. Moreover, our study identifies a pivotal role for NAP1L1 in myocardial fibrosis post injuries and inhibition of the function of NAP1L1 might be a therapeutic strategy for preventing heart failure.

## 2 RESULTS

### 2.1 NAP1L1 is upregulated during cardiac fibrosis and heart failure

**FIGURE 1:**
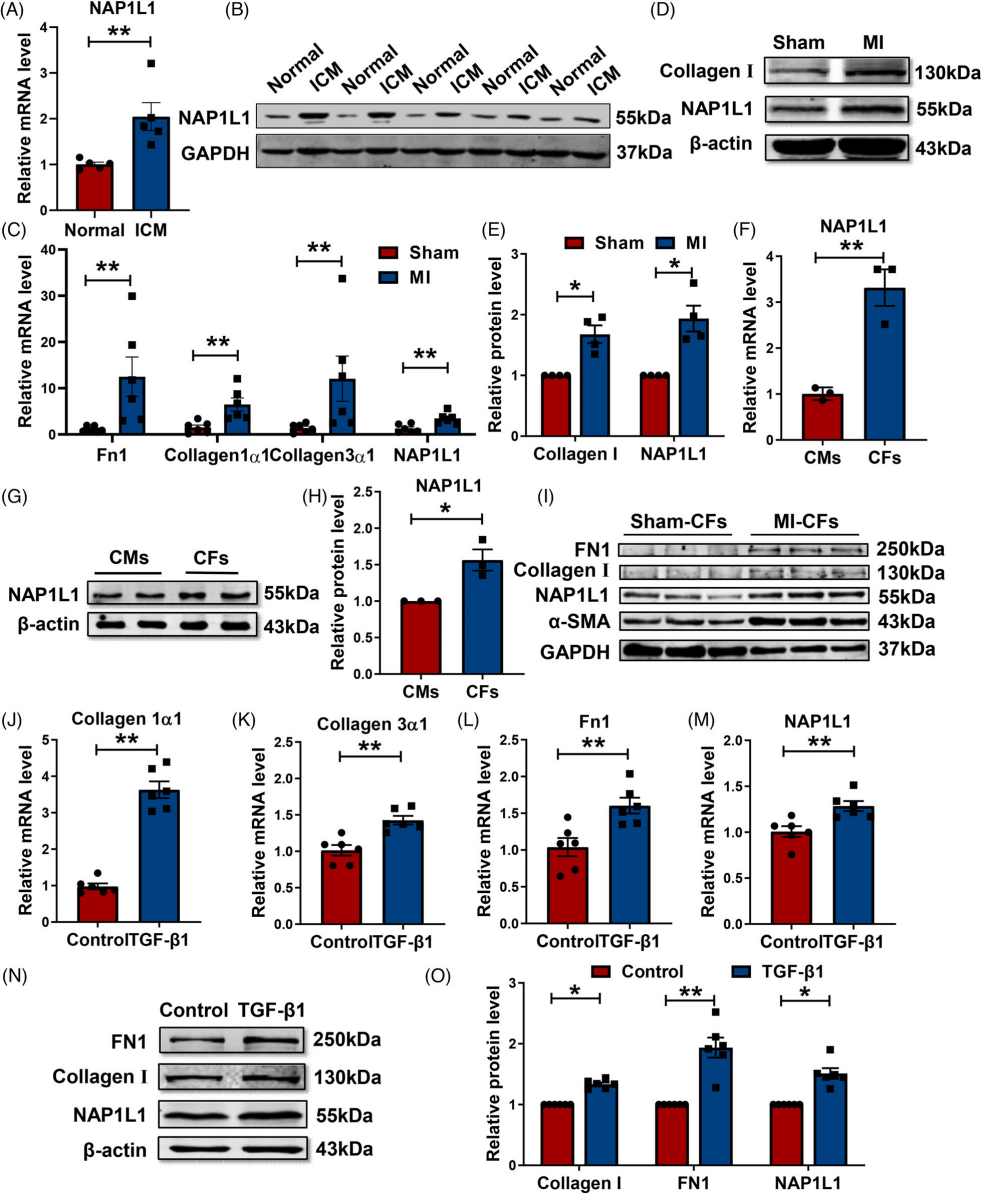
NAP1L1 was upregulated in heart failure, MI heart tissues, and TGF‐β1‐induced CFs. (A and B) Expression of NAP1L1 in normal and ischemic cardiomyopathy (ICM) patient hearts; *n* = 5, **p < 0.01. (C) The mRNA expression levels of Fn1, Collagen 1α1, Collagen 3α1, and NAP1L1 in Sham or after MI heart tissues; *n* = 6, ^**^ *p* < 0.01. (D and E) Western blots showed markedly increased expression of Collagen I and NAP1L1 in post‐MI cardiac tissue; *n* = 4, ^*^ *p* < 0.05. (F) Expression levels of NAP1L1 mRNA in CFs and CMs; *n* = 3, ^**^ *p* < 0.01 vs. CMs. (G and H) The protein expression level of NAP1L1 in CFs and CMs; *n* = 3, ^*^ *p* < 0.05 vs. CMs. (I) Western blotting was used to determine the expression of NAP1L1 in isolated cardiac fibroblasts of adult mice following MI. (J–M) The mRNA expression levels of Collagen 1α1, Collagen 3α1, Fn1, and NAP1L1 in TGF‐β1‐induced CFs; *n* = 6, ^**^ *p* < 0.01 vs. control. (N and O) Protein expression of Collagen I, FN1, and NAP1L1 after TGF‐β1 stimulation; *n* = 6, ^**^ *p* < 0.01, ^*^ *p* < 0.05 vs. control.

We first found NAP1L1 was dramatically upregulated in patients with ICM compared with normal hearts (Figures 1A and B). To detect the expression of NAP1L1 in MI‐induced cardiac fibrotic heart tissues. Mice were implanted with left anterior descending (LAD) ligation, and after 4 weeks of surgery, the heart tissue infarct border zone was isolated to detect the expression of NAP1L1 and fibrosis‐associated genes. The expressions of Fibronectin 1 (Fn1), Collagen 1α1, Collagen 3α1, and NAP1L1 were dramatically upregulated post‐MI at the mRNA level compared to the Sham group (Figure 1C). Similarly, fibrotic tissues had higher protein levels of NAP1L1 (Figures 1D and E). To examine the distribution of NAP1L1 in different cells, we detected the protein and mRNA levels of NAP1L1 in two major cell lines in the heart, CFs, and cardiomyocytes (CMs), respectively. As shown in Figures 1F–H, Cells from CFs expressed NAP1L1 at a higher level than those from CMs. Consistent with the results of heart tissues, CFs were isolated from adult mice post‐MI after 28 days, and NAP1L1 expression was also considerably upregulated (Figure 1I). Thus, in the following study, TGF‐β1 was used to induce a fibrosis model in vitro experiment. The expression of NAP1L1 was also obviously upregulated in TGF‐β1‐induced CFs (Figures 1J–O). These results showed that NAP1L1 was upregulated in myocardial fibrosis, and mainly located in CFs.

### 2.2 Overexpression of NAP1L1 results in fibrogenesis in CFs

**FIGURE 2:**
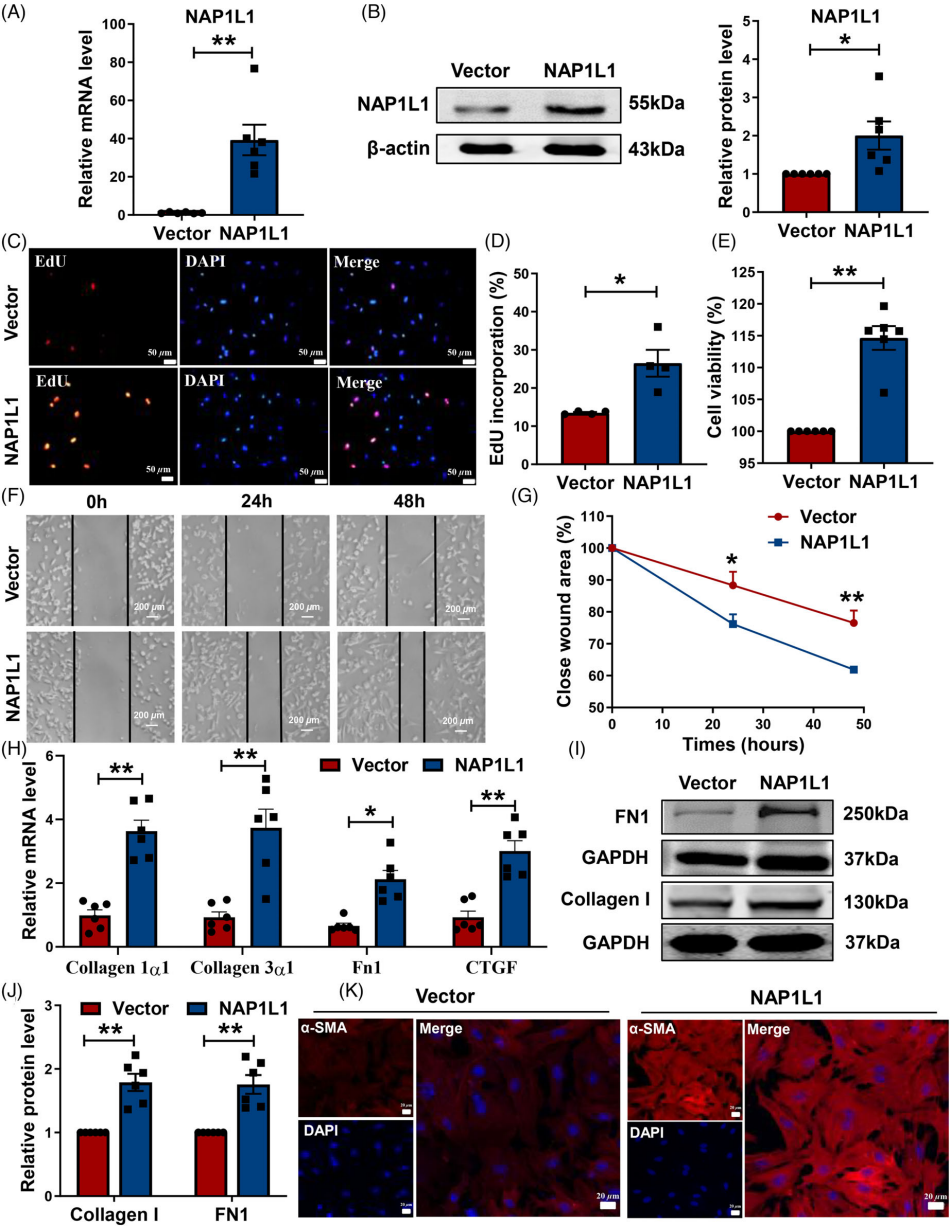
NAP1L1 promotes CFs proliferation, migration, and transition. (A and B) qRT‐PCR and Western blotting analysis of relative NAP1L1 expression in CFs treated with NAP1L1 overexpression plasmid or vector; *n* = 6, ^*^ *p* < 0.05, ^**^ *p* < 0.01. (C and D) The EdU assay showed the cell proliferation rate of CFs; bar = 50 μm; *n* = 4, ^*^ *p* < 0.05 vs. vector. (E) Cell viability by MTT assay; *n* = 6, ^**^ *p* < 0.01 vs. vector. (F and G) Cell migration assessed by wound healing assay; bar = 200 μm, *n* = 5, ^**^ *p* < 0.01, ^*^ *p* < 0.05 vs. vector. (H) The relative mRNA expression levels of Collagen 1α1, Collagen 3α1, Fn1, and CTGF after overexpressing NAP1L1; *n* = 6, ^**^ *p* < 0.01, ^*^ *p* < 0.05 vs. vector. (I and J) The protein level of Collagen I and FN1 after simultaneous overexpression of NAP1L1; *n* = 6, ^**^ *p* < 0.01 vs. vector. (K) Immunofluorescence assay was used to detect the expression level of α‐SMA; bar = 20 μm.

To identify whether NAP1L1 may affect cardiac remodeling, we detected the CFs proliferation, migration, and collagen synthesis after overexpression of NAP1L1. We first verified the overexpression efficiency in fibroblasts (Figures 2A and B). In the 5‐ethynyl‐2′‐deoxyuridine (EdU) assay, we observed a striking proliferation of fibroblasts upon overexpression of NAP1L1 (Figures 2C–D). 3‐(4,5‐Dimethylthiazol‐2‐yl)−2,5‐diphenyltetrazolium bromide (MTT) assay revealed that overexpression of NAP1L1 promotes the viability rates of CFs compared to the vector group (Figure 2E). Meanwhile, cell migration ability was also increased in the NAP1L1 overexpression CFs (Figures 2F and G). In addition, qRT‐PCR assays showed that overexpression of NAP1L1 in CFs can remarkably upregulate the mRNA expression levels of these fibrosis‐related factors compared to the vector group (Figure 2H). Overexpression of NAP1L1 increases the protein expression levels of FN1 and Collagen I in CFs (Figures 2I and J). CFs can transdifferentiate into myofibroblasts in the fibrosis process to secrete more collagen, we then detected the α‐SMA level, a marker of myofibroblast by immunofluorescence assay. As demonstrated in Figure 2K, the fluorescence intensity of α‐SMA was markedly enhanced after NAP1L1 overexpression. The above results suggested that forced expression of NAP1L1 aggravated myocardial fibrosis through reduced ECM deposition and fibroblast differentiation.

### 2.3 NAP1L1 knockdown alleviates TGF‐β1‐induced proliferation, migration, and differentiation in CFs

**FIGURE 3:**
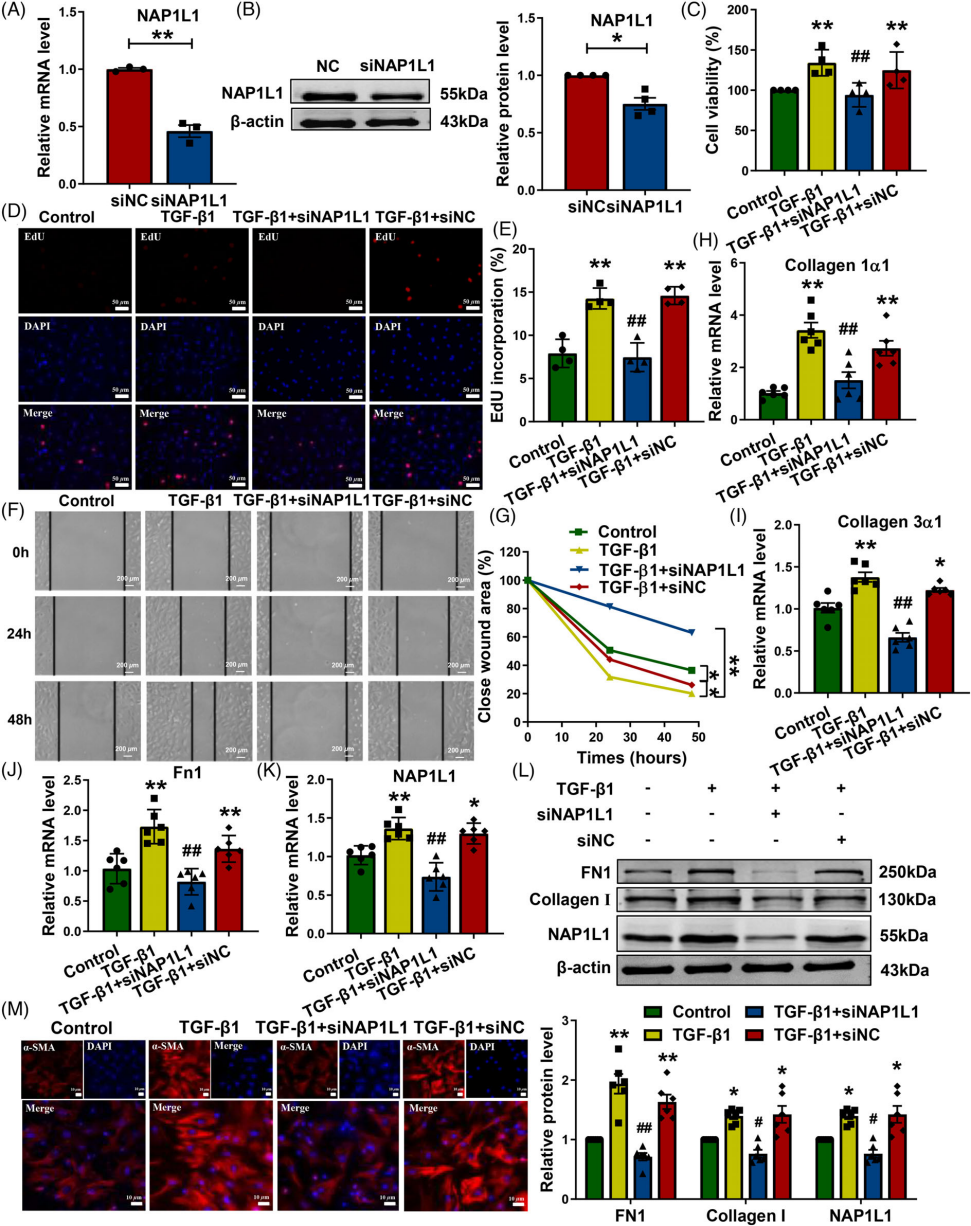
Silencing NAP1L1 inhibits CFs proliferation, migration, and transition induced by TGF‐β1. (A and B) Verification of siNAP1L1 knockdown efficiency at the mRNA level (left, *n* = 3) and protein level (right, *n* = 4) in CFs. ^*^ *p* < 0.05, ^**^ *p* < 0.01 vs. siNC. (C) Cell viability by MTT assay; *n* = 4, ^**^ *p* < 0.01 vs. control, ^##^ *p* < 0.01 vs. TGF‐β1. (D and E) Cell proliferation rate by EdU assays; bar = 50 μm, *n* = 4, ^**^ *p* < 0.01 vs. control, ^##^ *p* < 0.01 vs. TGF‐β1. (F and G) The wound healing assay assesses cell migration; bar = 200 μm, *n* = 8, ^*^ *p* < 0.05, ^**^ *p* < 0.01. (H–K) The mRNA expression levels of Collagen 1α1, Collagen 3α1, Fn1, and NAP1L1 in TGF‐β1‐induced CFs transfected with siNAP1L1; *n* = 6, ^**^ *p* < 0.01, ^*^ *p* < 0.05 vs. control, ^##^ *p* < 0.01 vs. TGF‐β1. (L) Silencing of NAP1L1 reduced the protein expression of FN1 and Collagen I that was induced by TGF‐β1; *n* = 6, ^*^ *p* < 0.05, ^**^ *p* < 0.01 vs. control, ^#^ *p* < 0.05, ^##^ *p* < 0.01 vs. TGF‐β1. (M) Immunofluorescence analysis showed that siNAP1L1 inhibited TGF‐β1‐induced the transition of fibroblasts into myofibroblasts; bar = 10 μm, *n* = 5.

Next, we constructed a small interfering RNA (siNAP1L1) to inhibit NAP1L1 endogenous expression in CFs (Figure 3A and B). As demonstrated in Figure 3C, the viability of CFs was significantly upregulated after TGF‐β1‐induced, and this change was substantially attenuated by siNAP1L1. The EdU assay revealed that siNAP1L1 notably inhibited CF proliferation induced by TGF‐β1 (Figures 3D and E). In addition, our team determined whether NAP1L1 affects fibroblast migration by using wound‐healing assays. As illustrated in Figures 3F and G, NAP1L1 knockdown can inhibit cell migration and abate wound healing induced by TGF‐β1. Moreover, qRT‐PCR revealed that silencing of NAP1L1 inhibits Collagen 1α1, Collagen 3α1, and Fn1 expression in TGF‐β1‐induced CFs (Figures 3H–K). At the same time, silencing NAP1L1 also reduced the expression of FN1 and Collagen I at the protein level in CFs stimulated by TGF‐β1 (Figure 3L). Immunofluorescence experiments revealed that the fluorescence intensity of α‐SMA was decreased in the siNAP1L1 group, and the knockdown of NAP1L1 reduced the trans‐differentiation of fibroblasts into myofibroblasts (Figure 3M).

### 2.4 Deficient NAP1L1 inhibits fibrosis and enhances cardiac function in MI mice

**FIGURE 4:**
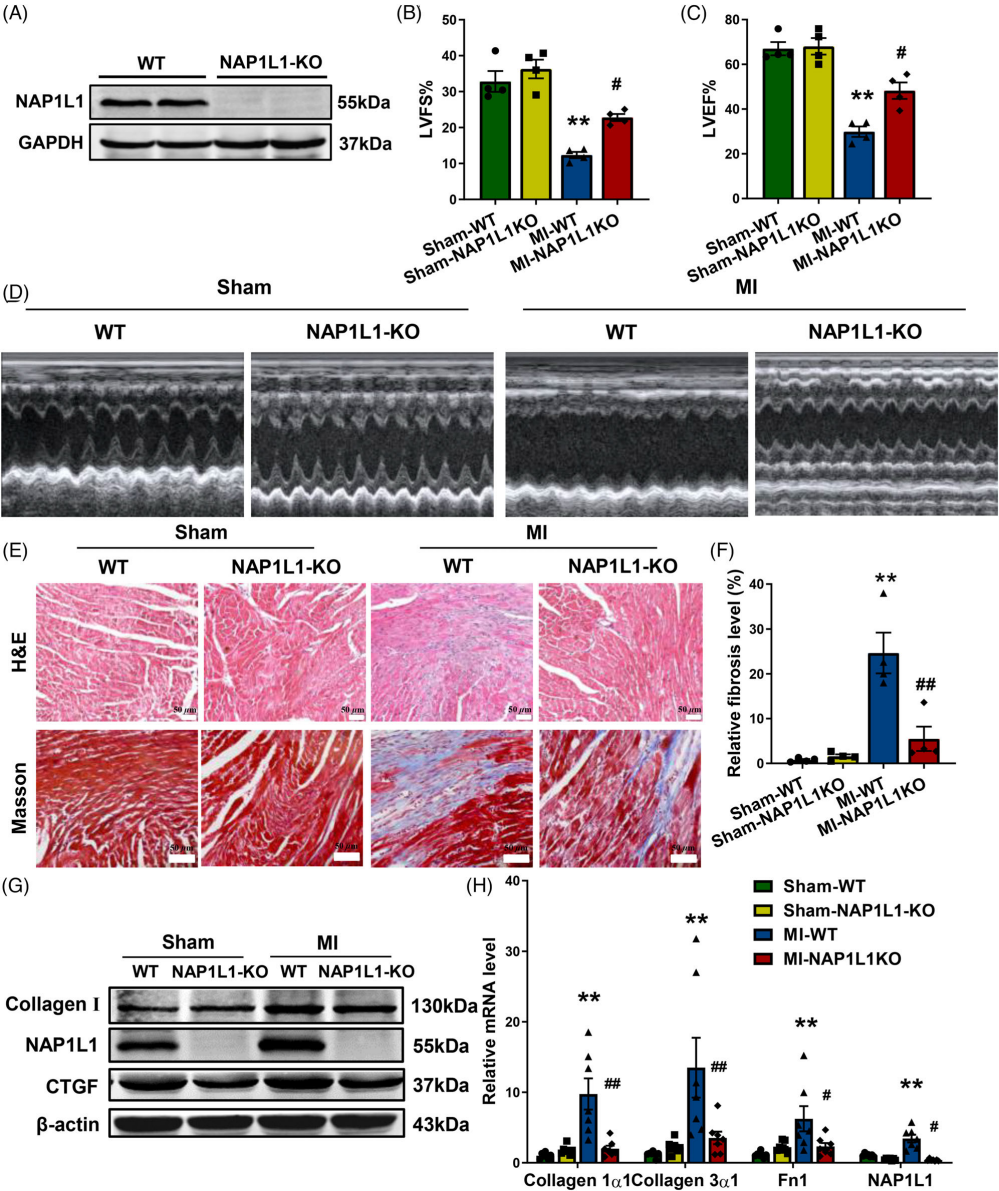
NAP1L1‐KO can reduce cardiac fibrosis in MI mice. (A) NAP1L1 expression level in NAP1L1 knockout mice was confirmed by WB. (B–D) Echocardiographic measurement of left ventricular ejection fraction (LVEF) (B), left ventricular fractional shortening (LVFS) (C); *n* = 4, ^**^ *p* < 0.01 vs. Sham‐WT; ^#^ *p* < 0.05 vs. MI‐WT. (D) Images of representative echocardiography. (E and F) Representative images and quantification of heart sections stained with Masson's trichrome and H&E staining results of mice heart tissues; bar = 50 μm, *n* = 4, ^**^ *p* < 0.01 vs. Sham‐WT; ^##^ *p* < 0.01 vs. MI‐WT. (G) Western blot assays were performed to detect the protein levels of Collagen I, NAP1L1, and CTGF. (H) The mRNA expression level of Collagen 1α1, Collagen 3α1, Fn1, and NAP1L1 by qRT‐PCR; *n* = 7, ^**^ *p* < 0.01 vs. Sham‐WT; ^#^ *p* < 0.05, ^##^ *p* < 0.01 vs. MI‐WT.

For further clarification of the role of NAP1L1 in cardiac fibrosis, we constructed NAP1L1 knockout (NAP1L1‐KO) mice which were established in the C57BL/6 background. Western blotting results verified the knockout efficiency in the heart (Figure 4A). Mice cardiac infarction models were then constructed in 6−8 weeks old homozygous KO mice. Ultrasonic parameters demonstrated that the ejection fraction (EF) and fractional shortening (FS) were greatly increased in NAP1L1‐KO mice compared with WT mice after MI surgery (Figures 4B–D). Meanwhile, hearts were harvested from different groups for HE staining and Masson's trichrome staining. MI‐NAP1L1‐KO hearts showed smaller‐scale fibrosis in the infarct zone compared with MI‐WT (Figures 4E and F). However, NAP1L1 knockout exerts no effect on the excessive accumulation of ECM compared with WT mice. In addition, Western blotting results illustrated that Collagen I and CTGF protein expression levels were upregulated in MI mice, whereas NAP1L1 knockout counteract these fibrotic effects (Figure 4G). Moreover, fibrosis‐related factors such as Collagen 1α1, Collagen 3α1, and Fn1 were also downregulated in MI‐NAP1L1‐KO mice when compared with MI‐WT mice (Figure 4H). As shown above, the knockout of NAP1L1 can alleviate cardiac fibrosis levels and improve cardiac function following MI.

### 2.5 NAP1L1 inhibits YAP1 ubiquitination and degradation

**FIGURE 5:**
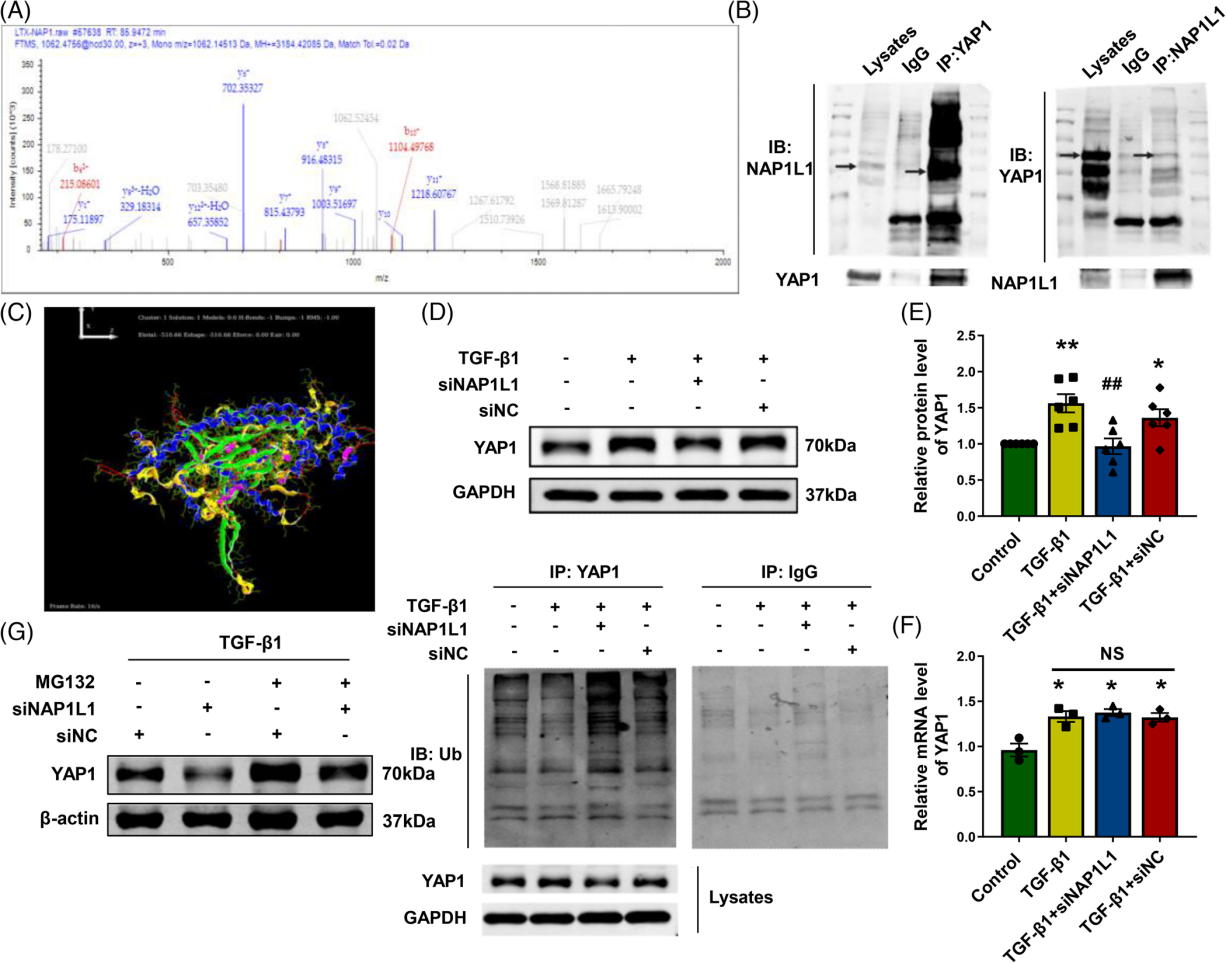
NAP1L1 targets YAP1 for ubiquitination and degradation. (A and B) CFs were transfected with the indicated plasmid and then lysed for the Co‐IP assay and LC‐MS/MS analysis using anti‐Flag beads to detect YAP1 and NAP1L1 binding. (C) Using HEX 8.0.0 software to study molecular docking. (D and E) Silencing NAP1L1 reverses the increase of YAP1 induced by TGF‐β1 via Western blot; *n* = 6, ^*^ *p* < 0.05, ^**^ *p* < 0.01 vs. control, ^##^ *p* < 0.01 vs. TGF‐β1. (F) qRT‐PCR validated that mRNA expression of YAP1; *n* = 6, ^*^ *p* < 0.05 vs. control. (G) The TGF‐β1‐induced cells were treated with proteasome inhibitor MG132 to observe the degradation of YAP1 protein after the knockdown of NAP1L1. (H) Representative immunoblots of the YAP1 ubiquitination levels in CFs transfected with the NAP1L1 siRNA.

We first used HEX 8.0.0 software and carried out Co‐immunoprecipitation (Co‐IP) combined with LC–MS/MS analysis to identify the proteins that interact with NAP1L1. We found that NAP1L1 can bind to YAP1 (Figures 5A–C). In recent studies, YAP1 has been shown to increase CFs proliferation and differentiation and plays a pivotal role in MI.^18^ We then detected if NAP1L1 could regulate cardiac fibrosis by interacting with YAP1. Western blotting results indicated that siNAP1L1 can reverse the TGF‐β1‐induced upregulation of YAP1 while qRT‐PCR results showed no effect on YAP1 mRNA (Figures 5D–F), indicating that NAP1L1 might regulate YAP1 of posttranscriptional modifications under TGF‐β1 stimulation. As demonstrated in Figure 5G, treatment with the 26S proteasome inhibitor MG132 causes accumulation of YAP1 but NAP1L1 reversed the effect of YAP1 degradation. It is suggested that during the cardiac fibrosis process, NAP1L1 is the involvement of the ubiquitin‐26S proteasome pathway to regulate YAP1 degradation. Furthermore, the Co‐IP assay also showed that silencing NAP1L1 can promote the ubiquitination of YAP1 under TGF‐β1 stimulation (Figure 5H).

### 2.6 YAP1 is essential for the antifibrotic effect of NAP1L1 silencing in TGF‐β1‐induced CFs

**FIGURE 6:**
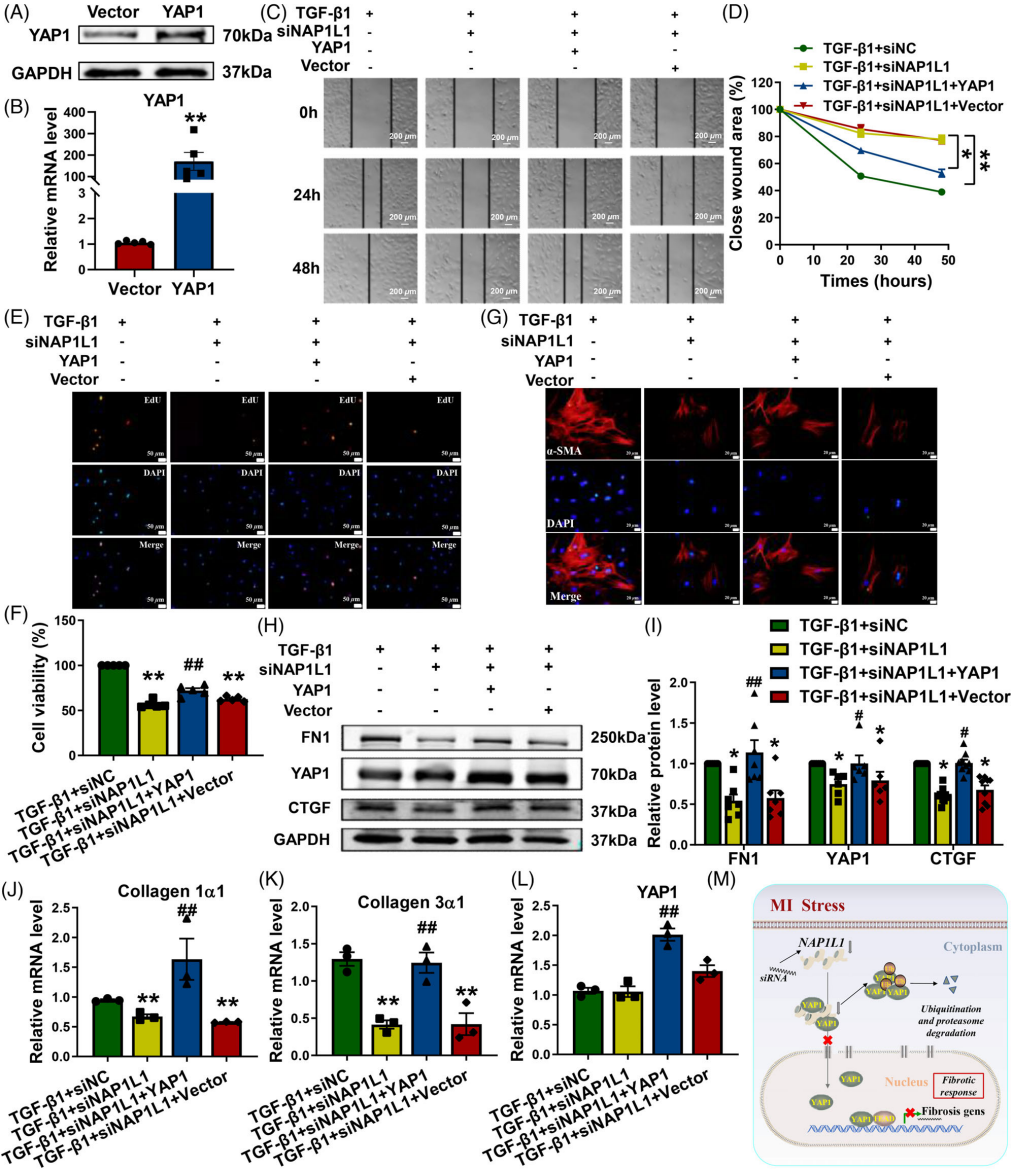
YAP1 reverses the effect of NAP1L1 in CFs. (A and B) The overexpression efficiency of YAP1 in CFs; *n* = 5, ^**^ *p* < 0.01 vs. control. (C and D) Quantification of scratch wound healing assays; bar = 200 μm; *n* = 6. ^*^ *p* < 0.05, ^**^ *p* < 0.01. (E) EdU detected the proliferation of CFs; bar = 50 μm; *n* = 3. (F) Knockdown NAP1L1 inhibited the viability of TGF‐β1‐induced CFs and this effect was abolished after overexpression of YAP1; *n* = 5, ^**^ *p* < 0.01 vs. TGF‐β1+siNC, ^##^ *p* < 0.01 vs. TGF‐β1+siNAP1L1. (G) Immunofluorescence staining of α‐SMA showed silencing of NAP1L1 slower YAP1 induced the fibroblast–myofibroblast transition after TGF‐β1 induction; bar = 20 μm, *n* = 6. (H and I) Western blot detected the expression levels of FN1, YAP1, and CTGF in CFs stimulated with TGF‐β1; *n* = 6, ^*^ *p* < 0.05 vs. TGF‐β1+siNC, ^##^ *p* < 0.01 vs. TGF‐β1+siNAP1L1. (J–L) Analysis of the relative mRNA expression of Collagen 1α1, Collagen 3α1, and YAP1 in TGF‐β1‐induced CFs cotransfected with YAP1 overexpression plasmid and NAP1L1 siRNA; *n* = 3, ^**^ *p* < 0.01 vs. TGF‐β1+siNC. ^##^ *p* < 0.01 vs. TGF‐β1+siNAP1L1. (M) Under conditions of fibrotic stimulus, knockdown of NAP1L1 reverses cardiac fibrosis through the regulation of YAP1 ubiquitination and degradation.

Further demonstrating the role of YAP1 in the effect of siNAP1L1 during fibrosis, we performed a rescue experiment. Therefore, we transfected YAP1 overexpression plasmid and siNAP1L simultaneously and detected the expression of YAP1 plasmid efficiency (Figures 6A and B). At the same time, as shown in Figures 6C–E, siNAP1L1 could strikingly attenuate TGF‐β1‐induced cell proliferation and ablated wound healing, whereas overexpression of YAP1 abolished the antifibrotic action of siNAP1L1. As illustrated in Figure 6F, the viability of TGF‐β1‐induced CFs was considerably decreased after siNAP1L1 transfection, and this change was markedly attenuated by overexpression of YAP1. Immunofluorescence depicted that knockdown NAP1L1 decreased the level of α‐SMA and suppressed myofibroblast differentiation, but the forced expression of YAP1 alleviated this effect (Figure 6G). Moreover, Western blotting and qRT‐PCR results revealed that overexpression of YAP1 increases fibrosis‐related factors expression, which was inhibited by NAP1L1 siRNA (Figure 6H–L). These data show that siNAP1L1 reverses the TGF‐β1‐induced fibrosis through the regulation of YAP1 ubiquitination and degradation.

## 3 DISCUSSION

We characterized for the first time the cardiac injury properties and the underlying mechanisms of NAP1L1 in MI mice and TGF‐β1‐induced CFs. First, we found that NAP1L1 was markedly upregulated in a mouse model of MI or TGF‐β1‐induced cardiac fibrosis in vitro. Knockdown of NAP1L1 attenuated cardiac fibrosis, whereas overexpression of NAP1L1 promoted fibroblasts proliferation, migration, and transition. Furthermore, we also demonstrated that NAP1L1‐KO mice markedly improved cardiac functions post‐MI and protect against pathological cardiac remodeling. Mechanistically, under conditions of fibrotic stimulus, knockdown of NAP1L1 reverses cardiac fibrosis through the regulation of YAP1 ubiquitination and degradation (Figure 6M).

Myocardial fibrosis post‐MI plays a crucial role in the development of heart failure and the appearance of remodeling and cardiac dysfunction eventually develops into heart failure.^19^ Continuous or repeated aggravation of myocardial ischemia and hypoxia will cause myocardial fibrosis, which is characterized by excessive ECM deposition.^20^ In recent years, lots of regulating factors were reported involved in the cardiac fibrosis process. Shih et al.^2^ found that endoplasmic reticulum protein TXNDC5 promotes cardiac fibrosis through folding ECM protein and CFs activation. Tripartite motif protein 72 (TRIM72), a protein containing a tripartite motif, can regulate cardiac fibrosis by targeting fibroblast proliferation and transition to myofibroblast.^21^ In vitro and in vivo studies showed that knocking down of NAP1L1 inhibited fibroblast‐to‐myofibroblast transition, reduced ECM deposition, and MI mice showed improved cardiac function.

The NAP1 gene family shares a common substructure, the NAP domain, and contains at least five members: NAP1L1–5.^22^ Among these NAP1 gene families exhibit a strong brain expression, whereas NAP1L1 and NAP1L4 are ubiquitously expressed in many different organs.^23^ NAP1L1 can function as a histone chaperon and chromatin assembly, which reveals that NAP1L1 strongly affects histone shuttling, cell‐cycle regulation, and cell proliferation.^10^ NAP1L1 is mainly located in microtubules. Previous work has indicated that NAP1L1 is a potential oncogene in many cancers.^13^, ^24^ Clinical research also showed that NAP1L1 was upregulated in colon cancer patients' serum and was a novel predictive and prognostic biomarker for colon cancer and lung adenocarcinoma.^25^, ^26^ However, its role in cardiovascular diseases remains unclear. Our study reveals for the first time that NAP1L1 is an essential profibrotic factor that contributes to the progression of fibrosis during MI. It was found in our research that NAP1L1 was upregulated in the tissues of fibrotic heart and knockout of NAP1L1 can robustly reduce heart fibrosis area after MI. Besides, we also found that NAP1L1 can promote CF proliferation, migration, and transition processes. Yan et al.^27^ indicated that NAP1L1 promotes iPSC proliferation by activating AKT or ERK signaling and Li et al.^28^ also found that siNAP1L1 can promote DMSO‐induced embryonic carcinoma cells differentiation into CMs. However, scarce reports are forcing on the role and the mechanism of NAP1L1 in fibroblast proliferation, especially in CFs. We then assumed that NAP1L1 exerted its profibrotic effect and regulate CFs proliferation and migration through some proteins or signaling pathways.

To corroborate the hypothesis, we used HEX 8.0.0 software and Co‐IP with LC–MS/MS analysis to identify the protein that interacts with NAP1L1. Among these, we focused on YAP1, which showed a high binding coefficient with NAP1L1, and Co‐IP was performed to confirm the binding of NAP1L1 with the YAP1 protein. As shown, siNAP1L1 can decrease the protein expression but not YAP1 mRNA expression, indicating that NAP1L1 may regulate the posttranscriptional modifications process of YAP1. Further research found that silencing NAP1L1 can promote the ubiquitination of YAP1 in CFs. Evidence showed that E3 ubiquitin ligase SCF (beta‐TRCP) could catalyze YAP1 ubiquitination, leading to YAP degradation.^29^ Besides, FAT10, a ubiquitin‐like protein, is capable of regulating ubiquitination and degradation of YAP1.^30^ Moreover, FAT10, a ubiquitin‐like protein, is capable of regulating ubiquitination and degradation of YAP1. Thus, which kind of E3 ubiquitin ligase was involved in NAP1L1‐regulated YAP1 ubiquitination needs further discovery. Besides, phosphorylation is the most common posttranscriptional type of YAP1.^31^ NAP1L1 effects on YAP1 phosphorylation level also need further investigation.

YAP1, a multifunctional intracellular connexin, and transcription coactivator plays a central hub in the Hippo signaling pathway and the proliferation of tumor cells.^32^ It is becoming increasingly evident that YAP1 is considered to promote tumor cell proliferation and inhibit apoptosis protein.^17^ In addition, YAP1 activation developmentally regulates embryonic heart size and promotes the proliferation of neonatal or adult CMs and the regenerative capacity of neonatal mouse cardiac.^33^ However, the specific mechanism that regulates YAP1 and activates it is unclear yet. This study used Co‐IP in combination with LC‐MS/MS analysis to prove that YAP1 binds to NAP1L1. Since the degradation of YAP1 is reduced after the use of protease inhibitor MG132 and silencing NAP1L1 reversed this effect, it indicates that siNAP1L1 degrades YAP1 through a proteasome‐dependent pathway. Further, investigations are required to probe whether NAP1L1 acts on other proteins that affect the progression of cardiac fibrosis.

Although research on NAP1L1 in fibrosis has achieved some progress and highlights an important factor regulation of fibrosis, it also has certain limitations. We only detected NAP1L1 expression level in mice fibrosis cardiac tissues, but fail to detect its expression level in patients’ serum. It is a limited in‐depth exploration of NAP1L1 clinical applications. In addition, our current study is also limited by a lack of NAP1L1 conditional knockout mice, for specifically investigating NAP1L1 specificity functions in fibroblasts and ruling out nonfibroblast cell effect in vivo. Another consideration is that we did not verify the specific binding site of NAP1L1 that regulates YAP1 ubiquitination and degradation, limiting our deep explorations of the relationship between NAP1L1 and YAP1. Notwithstanding its limitation, this study does suggest that NAP1L1 is of no benefit to cardiac function. We will further explore the mechanism in future research.

In summary, this study sheds light on a novel regulatory role of NAP1L1 in fibrosis during MI. We demonstrated that NAP1L1 is a profibrotic factor that inhibits YAP1 ubiquitination and degradation to regulate cardiac fibrosis. Therefore, it is possible to speculate that suppression of NAP1L1 is a potential intervention treatment target for MI, providing a novel idea.

## 4 METHODS AND MATERIALS

### 4.1 Patients and tissue specimens

Research on human heart tissue got approval from the Medical Ethics Committee of Zhongnan Hospital of Wuhan University (No. 2022146K) and adhered to all relevant ethical guidelines. Transplant patients and families of donors provided informed consent for tissue collection. A failing sample was obtained from patients with ischemic heart failure (*n* = 5), while a nonfailing sample was obtained from healthy donors without a history of heart failure (*n* = 5).

### 4.2 Mice

We used heterozygous recombinant NAP1L1 knockout mice. The knockout mice were generated by Cyagen Biosciences Inc. The homozygous mice were produced by intercrossing heterozygous mice. TaKaRa MiniBEST Universal Genomic DNA Extraction kit (Version 5.0) was used to extract genomic DNA from 1‐month‐old mice toes for subsequent PCR genotyping. NAP1L1 knockout mice primers are shown below: (forward 1: 5′‐ GTCCTCTGTAAGTGGTCGTAGCC‐3′; forward 2: TGAATGTTGTATGGAAAGTGTGAC‐3′; reverse: 5′‐ GTACTAACCGAAACTTCATCCTCC‐3′). An age‐matched group of six to eight‐week‐old C57BL/6 mice weighed 18–23 g was obtained from Changsheng Biotechnology.

### 4.3 MI model and echocardiography

To induce cardiac fibrosis, mice MI model was established as previously described.^34^ Briefly, male mice in the supine position were sedated by sodium pentobarbitone (40 mg/kg, i.p.) and xylazine (12.5 mg/kg, i.p.) in the supine position. Through a lateral thoracotomy, the hearts were exposed, and a silk suture was used to tie the LAD permanently. As above, the manipulation of Sham‐operated mice was similar without ligation of their LADs. We performed a two‐dimensional M‐mode echocardiogram four weeks after MI with VINNO6 (VINNO Technology). Left ventricle FS and EF were measured.

### 4.4 Histology and immunohistochemistry

Hearts were harvested and fixed in 4% paraformaldehyde. After 48 h, dehydration was operated and then heart tissues were embedded in paraffin. Following sectioning, tissues were stained with Masson stain and immunohistochemically analyzed. The Masson staining procedure followed the manufacturer's instructions for the Masson's Trichrome Stain Kit (Solarbio; G1340). For immunostaining, the sections were deparaffinized and blocked for ten minutes with 3% H_2_O_2._ After three rinses in PBS, tissue sections then went through an antigen retrieval process and were blocked for 1 h with 50% goat serum. After that, primary antibodies α‐SMA (Affinity; AF1032); NAP1L1 (Abcam; ab33076) were added and incubated at 4°C overnight. The next day, sections were incubated for 30 min at 37°C with a secondary antibody (ZsBio; ZB‐2301) after washing with PBS. Three rinses in PBS used DAB for color development (ZsBio; ZLI‐9018). At last, nuclear staining was completed with Hematoxylin, followed by residence seals for further detection.

### 4.5 Cell culture and transfection

Heart tissues from neonatal mice were gained from 1−3 days‐old mice, including CFs and CMs. Mice hearts were extracted and suspended in a mixture of DMEM (Biological Industries, Israel) medium and 0.25% trypsin (3:2) and shaken for 12 h at 4°C. Then added 5 mL of complete medium was to neutralize and discarded the supernatants. As the collagenase II solution was introduced, 7 mL was added at a time until all heart tissues were digested. After centrifugation, then cell suspensions were resuspended in a complete medium. Cell culture plates were plated with the suspension, and the supernatants were removed for 45 min and repeated twice. CMs supernatants from the culture were transferred to another culture plate, where they were incubated for 48 h at 37°C with 5% CO_2_. In addition, NAP1L1‐targeting plasmids and siRNA (Sigma; CAGACUUUGAAAUUGGUCAdTdT) were transfected into the CFs. YAP1 and NAP1L1 plasmids were inserted into the pcDNA3.1+ vector. Human TGF‐β1 (10 ng/mL; PeproTech) was administered to CFs for a duration of 48 h.

### 4.6 Migration assay

The wound healing assay was conducted to determine the capacity of cell migration, following the previously described method.^35^ Briefly, the cell were cultured in six‐well plates for one night, and the following day a wound was made to assess the migratory capacity of the CFs. After transfection, using a p200 pipet tip to create a straight line, some cells were scraped. Images of cells were taken every 24 h using a Nikon Ts100 microscope (Nikon). The average area of wound closure was quantified by ImageJ.

### 4.7 MTT assay

Cell viability was detected using MTT. Forty‐eight hours after transfection, CFs were seeded into 96‐well plates and 20 μL MTT (0.5 mg/L) was added later and incubated together for 4 h at 37°C. Afterward, the medium was taken out and 200 μL of DMSO was added to dissolve MTT formazan was dissolved in 200 μL of DMSO. The absorbance was then recorded at a wavelength of 490 nm.

### 4.8 EdU assay

EdU fluorescence staining was applied to determine the proliferation of CFs following the manufacturer's instructions (RiboBio).

### 4.9 Immunofluorescence analysis

Cells were seeded in a 24‐well plate with glass slides in the bottom. After transfection, cells were washed three times by PBS and cold methanol used for fixing was added for 30 min. After permeabilization with 0.1% Triton‐X100 for 1 h, goat serum was added and incubated for 40 min. Afterward, incubated cells with primary antibody α‐SMA (Abcam; ab7817); NAP1L1 (Abcam; ab33076) overnight at 4°C. Secondary antibodies were added the next day 1 h after washing with PBS. Finally, the nuclei were stained by DAPI. Results were observed under a confocal microscope (ZEISS).

### 4.10 Western blotting

Cells and tissue proteins were extracted with RIPA supplemented with protease and phosphatase inhibitors (Roche, Basel, Switzerland). SDS‐PAGE gels with 10% protein loading were used to separate 20−60 μg of proteins, which were transferred to NC membranes in the next step. Membranes were then blocked with 5% skim milk for an hour. Following that, membranes were incubated overnight at 4°C with the first antibodies and secondary antibodies successively and scanned by Odyssey Imaging System (Odyssey CLX, Biosciences), which presented results at last. This experiment used antibodies including NAP1L1 (Abcam; ab174836); Collagen I (WanLei; WL0088); Fibronectin (Proteintech; 15613‐1‐AP); α‐SMA (Abcam; ab7871); CTGF (Proteintech; 25474‐1‐AP); β‐actin (Genscript; A00702); GAPDH (Proteintech; 10494‐1‐AP); YAP1 (Proteintech; 13584‐1‐AP); Ub (Santa; sc‐8017).

### 4.11 Quantitative real‐time PCR

The total RNA was extracted by TRIzol according to the standard protocol, followed by reverse transcription using All‐In‐One 5X RT MasterMix (Abm). Quantitative PCR was then performed using a LightCycler machine (ABI) and BlasTaqTM 2X qPCR MasterMix (Abm) to measure mRNA levels. The homo sapiens primers used in the study: NAP1L1 (forward: 5′‐CCTTTTCTTTTGACGGACCA‐3′; reverse: 5′‐GTATCGCCTCAGCATCATCA‐3′), Yap1 (forward: 5′‐GAGACACCATCAGCCAAAGC‐3′; reverse: 5′‐GCAGCCAACACAGACTCCAC‐3′), Collagen 1α1 (forward: 5′‐AAGAAGACATCCCTGAAGTCA‐3′; reverse: 5′‐TTGTGGCAGATACAGATCAAG‐3′), Collagen 3α1 (forward: 5′‐TTGGGATGCAGCCACCTTG‐3′; reverse: 5′‐CGCAAAGGACAGATCCTGAG‐3′), CTGF (forward: 5′‐GCTAAGTTCTGCGGGGTGT‐3′; reverse: 5′‐GTAATGGCAGGCACAGGTCT‐3′), Fn1 (forward: 5′‐ CGAGGTGACAGAGACCACAA‐3′; reverse: 5′‐GACACAACAATGCTCCCAGA‐3′), GAPDH (forward: 5′‐TCTACATGTTCCAGTATGACTC‐3′; reverse: 5′‐ACTCCACGACATACTCAGCACC‐3′); The homo sapiens primers used in the study: NAP1L1 (forward: 5′‐AAAGCACGTCAGCTAACTGTT‐3′; reverse: 5′‐TTGAGAGCATTCACTCGTCTTTT‐3′), GAPDH (forward: 5′‐GGAGCGAGATCCCTCCAAAAT‐3′; reverse: 5′‐GGCTGTTGTCATACTTCTCATGG‐3′).

### 4.12 Co‐immunoprecipitation

CFs were lysed and proteins were incubated with antibodies overnight at 4°C. Then, added 30 μL of pre‐prepared protein A/G magnetic beads (MCE) and incubated overnight at 4°C. Finally, Washed the beads with binding wash buffer 5 times before adding 30 μL elution buffer into the tube. Perform magnetic separation, and collect the supernatant following Western blotting analysis.

### 4.13 Statistics

There are at least four experiments represented as a mean and standard error of the mean (±SEM). ANOVA followed by Bonferroni post hoc tests were performed for comparisons among the multiple groups. A two‐tailed Student's *t*‐test was used for two groups. GraphPad Prism 7.0 was used to analyze the data. *p* < 0.05 was considered statistically significant.

## AUTHOR CONTRIBUTIONS

The project was conceived, designed, and edited by H. L., H. S., B. W, and R. Y. T. L. In this study, Z. N., X. L., T. Y., and L. L. designed the experiments, analyzed the data, and wrote the manuscript. J. F., B. X., D. B., and M. H. performed animal studies and analyzed the data. J. L., X. L., Q. W., and X. Y. carried out cellular and molecular biological experiments and executed data analysis. All authors have read and approved the final manuscript.

## CONFLICT OF INTEREST STATEMENT

All the authors have declared that there are no conflicts of interest.

## ETHICS STATEMENT AND CONSENT TO PARTICIPATE

The patient sample‐related experiments were approved by the Medical Ethics Committee of Zhongnan Hospital of Wuhan University (approval No. 2022146K, China) and compliant with all relevant ethical regulations. Written informed consent for research purposes was obtained from the patients. All animal experiments were approved by the Ethics Committee of Harbin Medical University (approval No. IRB3022621, China).

## Data Availability

All data and materials are available from the corresponding authors upon request once published.
